# Odor Recognition with a Spiking Neural Network for Bioelectronic Nose

**DOI:** 10.3390/s19050993

**Published:** 2019-02-26

**Authors:** Ming Li, Haibo Ruan, Yu Qi, Tiantian Guo, Ping Wang, Gang Pan

**Affiliations:** 1 College of Computer Science and Technology, Zhejiang University, Hangzhou 310027, China; lming@zju.edu.cn (M.L.); hbruan@zju.edu.cn (H.R.); 2 Department of Biomedical Engineering, Zhejiang University, Hangzhou 310027, China; 21315047@zju.edu.cn (T.G.); cnpwang@zju.edu.cn (P.W.); 3 State Key Lab of CAD&CG, Zhejiang University, Hangzhou 310027, China

**Keywords:** odor recognition, bioelectronic nose, spiking neural network

## Abstract

Electronic noses recognize odors using sensor arrays, and usually face difficulties for odor complicacy, while animals have their own biological sensory capabilities for various types of odors. By implanting electrodes into the olfactory bulb of mammalian animals, odors may be recognized by decoding the recorded neural signals, in order to construct a bioelectronic nose. This paper proposes a spiking neural network (SNN)-based odor recognition method from spike trains recorded by the implanted electrode array. The proposed SNN-based approach exploits rich timing information well in precise time points of spikes. To alleviate the overfitting problem, we design a new SNN learning method with a voltage-based regulation strategy. Experiments are carried out using spike train signals recorded from the main olfactory bulb in rats. Results show that our SNN-based approach achieves the state-of-the-art performance, compared with other methods. With the proposed voltage regulation strategy, it achieves about 15% improvement compared with a classical SNN model.

## 1. Introduction

Electronic noses usually use sensor arrays to recognize odors [1,2]. However, when facing many types of odors, traditional electronic nose requires a combination of different sensors, resulting in larger size and higher costs. In addition, sensors will be redesigned when given a new type of odor, which is difficult and expensive. Animals have natural sensory capabilities for recognizing various types of odors, and it is promising to provide new options for odor sensor design using biological components of animals. Bioelectronic noses use olfactory receptors [3] as sensing elements in the electronic smell systems [4,5]. Previous work has developed new biomaterials [6] for bioelectronic noses and obtained good results in odor detection tasks [7]. With the development of brain–machine interface (BMI) [8], biological neural activities can be in part understood with the recorded electronic neural signals, for example, motor imagery [9], gestures [10], and memory evaluation [11]. In this way, odor information could be decoded from neural signals recorded by implanting electrodes into the olfactory bulb of mammalian animals [12]. In virtue of the powerful biological olfactory system, bioelectronic noses are promising to provide fast response and high sensitivity, and will help to build cyborg intelligent systems that integrate machine and biological intelligence [13,14,15]. A big challenge for bioelectronic noses is how to effectively decode odors from neural signals.

Neural signals recorded from the olfactory bulb are temporal spike trains from multiple channels. For most machine learning algorithms requiring vector-based inputs, existing neural signal decoders transform spike trains into firing rates in time windows (bin-based approaches) as features [16]. The binned features can be easily processed by classical machine learning models. You et al. used a maximum likelihood estimation (MLE) method to recognize odors from spike trains recorded in rat olfactory bulbs [17]. Its key step was to calculate the difference of spike firing rate before and after odor stimulation as features, then build a Gaussian model with training data, and finally use maximum likelihood estimation to predict the input odor. Dong et al. used population vector similarity and support vector machine (SVM) for low concentration odor detection [12]. Ma et al. used the Kalman filter to decode the spike trains of neurons in the primary motor cortex to predict lower limb muscle activity [18]. The summary of these approaches is shown in Table 1. However, bin-based approaches ignore the rich timing information in spike trains, such as the firing time point of single spike and the time interval between neighboring spikes. It is a critical problem to exploit the rich information in spike timing for accurate and efficient neural signal decoding.

Spiking neural networks (SNNs) [19] mimic biological neural networks more closely by incorporating timing information in computing, which have proven to be effective in tasks such as sequence discrimination [20], object recognition [21], sound classification [22] and so on. The biologically plausible properties make SNN a promising option for neural decoding tasks. Instead of using binned features, SNN uses time points of spikes, which make it capable of processing precise timing information in spike trains [23]. Existing spiking neuron models include the Hodgkin–Huxley (HH) model [24], Leaky Integrate and Fire (LIF) model [25], Izhikevich model [26], etc. Learning algorithms of spiking neural networks can be divided into supervised learning algorithms and unsupervised learning ones [27]. Supervised learning algorithms include SpikeProp [28], Tempotron [29], Remote Supervised Method (ReSuMe) [30], Precise-Spike-Driven Synaptic Plasticity (PSD) [31], and so on. Unsupervised learning algorithms are relatively rare, and the most representative one is Spike Timing Dependent Plasticity (STDP) [32].

In this study, we propose a novel spiking neural network(SNN)-based neural signal decoder to recognize odors with spike train signals recorded from olfactory bulb in rats. Unlike most previous work that requires bin-based inputs, our approach exploits rich timing information in precise time points of spikes. To deal with a small amount of training data, we especially propose a new SNN learning method with a voltage-based regulation strategy (SNN-VR), in order to alleviate the overfitting problem during training. Experiments are carried out using spike train signals recorded from olfactory bulb in rats. Results show that, compared with bin-based odor decoders, the SNN approaches achieve better performance in two-class, three-class, and four-class odor recognition tasks. With the proposed voltage-based regulation strategy, the SNN-VR approach obtains about 15% improvement compared with the classical SNN model. SNN-VR demonstrates better generalization ability especially with small training sets.

## 2. Materials and Methods

**Figure 1:**
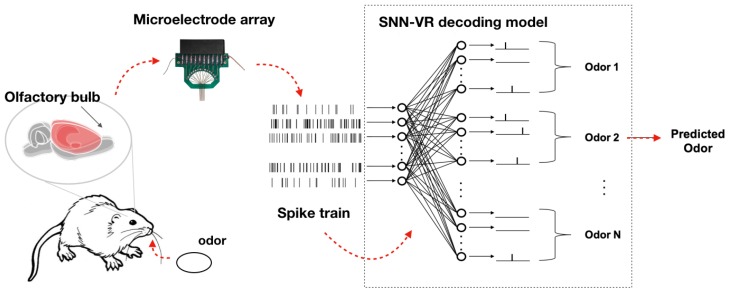
Odor recognition with spiking neural network.

In this section, we present the SNN-VR odor recognition method in detail. Firstly, we describe neural signal acquisition and processing procedure to record spike trains from olfactory bulb areas in rats. Then, we briefly introduce some typical classifiers using bin-based features. After that, we elaborate on how to effectively decode odor information from spike trains using SNNs, and give details of the proposed SNN-VR approach. The framework of the BMI-based bioelectronic nose is illustrated in Figure 1.

### 2.1. Data Acquisition

**Figure 2:**
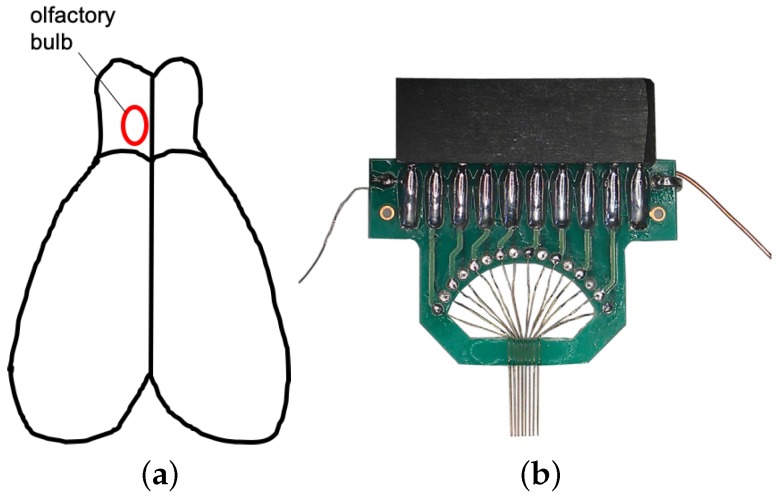
(**a**) illustration of rat brain and olfactory bulb area (red circle); (**b**) microelectrode array (8 × 2 microwires).

Spike train signals are recorded by implanting electrodes into the main olfactory bulb of rats [33]. Microelectrode arrays with 8 × 2 microwires were implanted vertically into the cell body layer (600–800 μm) of Mitral/Tufted (M/T) cells in rat olfactory bulbs to detect spike firing and field potential waveform as shown in Figure 2. Omiplex Neural Data Acquisition System (Plexon) was used to record electrophysiological signals and odor stimuli events. The sampling rate was 40 kHz. The spike waveform was obtained by filtering the raw data (250–3000 Hz, Butterworth) and the spikes were detected by double thresholds method. All procedures were carried out in strict accordance with a protocol approved by Zhejiang University Animal Care and Use Committee.

The odor stimuli were given by glass dishes containing the odor solutions in front of the rats’ nose. The concentration of all odor solutions was controlled to 10${}^{-3}$ mol/L. The neural signals were recorded during free breathing in anesthetized rats. According to the odor map [2], a total of four odors were used for analysis including octanol (odor-1), pentanal (odor-2), butyric acid (odor-3) and isopentyl acetate (odor-4), where the first three odors can cause strong neural response in olfactory bulb area while the fourth odor can only cause little neural response. The duration of odor stimulation was 4–5 s in each trial, and there were 5 trials a day for each odor. There was a 2-min interval between two consecutive trials and a 10-min interval between two kinds of odor stimuli. The signals were recorded continuously for 4 days to obtain enough data for decoding. Table 2 shows more detailed descriptions of the neural signal dataset.

In the dataset, there are a total of 80 samples of neural signals (20 samples per odor). The experiments are carried out using random test, that is, a total of 100 experimental trials are conducted and the average accuracy is presented. In an experimental trial, for each odor type, 16 samples are randomly selected for training, and the 4 remaining samples are used for testing.

### 2.2. Odor Recognition with Bin-Based Classifiers

With bin-based classifiers, we use spike counts in small time bins for each neuron as the features. We calculate the spike counts $c_{n_{i}}$ of each bin and combine these values into a feature vector. First, we select a bin of certain size and calculate spike counts of each bin as the feature $c_{n_{i}}\left(k\right)$, *n* represents the *n*-th neuron, *k* represents the odor type, and *i* is the index of the bin. The feature vector of each neuron is:

(1) $$x_{n}\left(k\right)=\left[c_{n_{1}}\left(k\right),c_{n_{2}}\left(k\right),\ldots ,c_{n_{M}}\left(k\right)\right],$$

where *M* represents the number of bins in the time period, taking a bin of 0.5 s and time period of 5.0 s as an example, *M* = 5.0 s/0.5 s = 10. Then the feature vectors of all neurons during each odor stimulation are combined into a new feature vector $x\left(k\right)=[x_{1}\left(k\right),x_{2}\left(k\right),\ldots ,x_{n}\left(k\right)]$.

After feature extraction, classifiers including decision tree (DT), k-nearest neighbors (KNN), linear discriminant analysis (LDA), maximum likelihood estimation (MLE) and supporting vector machine (SVM) can be used for odor recognition.

### 2.3. Odor Recognition with Spiking Neural Network

Spiking neural networks (SNNs) [19] are biologically plausible networks, which incorporate timing information in computing. Instead of using binned-based features, SNN can directly receive time points of spikes as inputs, therefore it preserves richer information in the firing time point of single spike and time interval between neighboring spikes [23].

In this study, we use a two-layer spiking neural network consisting of an input and an output layer for neural signals decoding. The number of neurons in the input layer is same as the recorded neurons, and there are groups of neurons (each group *m* representing one odor type) in output layer which fire zero or one spike. All neurons are fully connected.

#### 2.3.1. Spiking Neuron Model

In this study, we use Leaky Integrate and Fire (LIF) neuron model. The voltage of LIF neuron is determined by exponentially decayed synaptic current induced by input spikes. Each input spike from a presynaptic neuron *i* at time $t_{i}$ would induce a postsynaptic potential (PSP) on the postsynaptic neuron *j*, which can be described by the kernel function $K(t-t_{i})$:

(2) $$K\left(t-t_{i}\right)=V_{0}\left(exp\left[-\left(t-t_{i}\right)/\tau \right]-exp\left[-\left(t-t_{i}\right)/\tau_{s}\right]\right),$$

where $V_{0}$ normalizes the maximum value of PSP to 1. The parameters τ and $\tau_{s}$ are the decay time constants of membrane integration and synaptic currents respectively. $K(t-t_{i})$ is only valid for the input spikes before *t* ($t_{i}<t$) and becomes zero for the input spikes after *t* ($t_{i}>t$).

At time *t*, the membrane voltage of the postsynaptic neuron *j* is a weighted sum of all PSPs contributed by input spikes before time *t*:

(3) $$V_{j}\left(t\right)=\sum_{i}\omega_{ij}\sum_{t_{i}}K\left(t-t_{i}\right)+V_{rest},$$

where $\omega_{ij}$ represents the synaptic weight between the presynaptic neuron *i* and postsynaptic neuron *j*. When there is no input spike, the postsynaptic neuron *j* maintains the resting potential $V_{rest}$ (usually set to 0). A spike will be fired by the postsynaptic neuron *j* if $V_{j}\left(t\right)$ exceeds a certain threshold (usually set to 1).

#### 2.3.2. Tempotron

Tempotron is a classic learning rule for SNN in binary classification tasks [29]. Based on the Tempotron rule, a neuron fires a spike when it receives a spike pattern from the target class. The Tempotron learning rule updates synaptic weight $\omega_{ij}$ when errors occur. During iterative training, if a postsynaptic neuron *j* fires an error spike (spike fired by the group of neurons which represent wrong odor type), synaptic weight $\omega_{ij}$ would be suppressed according to its contribution to the firing of error spike. If a postsynaptic neuron *j* does not fire the expected spike (spike fired by the group of neurons which represent correct odor type), synaptic weight $\omega_{ij}$ would be strengthened according to its responsibility for the failure to fire the spike. To solve the problem of assigning the relative contribution of input spikes, Tempotron uses the following rule: if the expected spike does not occur, each synaptic weight $\omega_{ij}$ increases by a certain value $\Delta \omega_{i}$:

(4) $$\Delta \omega_{i}=\lambda \sum_{t_{i}<t_{max}}K\left(t_{max}-t_{i}\right),$$

where $t_{max}$ denotes the time when the postsynaptic potential $V_{j}\left(t\right)$ reaches its maximum value during the time period. The constant λ>0 specifies the maximum value of synaptic weight updating for each input spike. In contrast, if an error spike occurs, the synaptic weight $\omega_{ij}$ decreases by $\Delta \omega_{i}$.

#### 2.3.3. Tempotron with Voltage-Based Regulation Strategy

Since the Tempotron rule adjusts synaptic weights according to the classification errors, it does not update synaptic weights when training samples are correctly classified. However, when the training set is small, it usually causes that the membrane voltage thresholds of some neurons could be approached easily. When disturbances occur in spike trains, the neurons can fire mistakenly. In odor recognition tasks, the training data set is usually small. Therefore, we propose a new voltage-based regulation strategy called Tempotron-VR, to improve the training effectiveness.

Different from the Tempotron rule, the proposed Tempotron with voltage-based regulation strategy (Tempotron-VR) approach adopts a $\Delta \omega_{i}^{\prime }$ in synaptic weight updating. By this way, the maximal voltage value of neurons in the target group (representing correct odor type) will become larger during training process, so it will be prone to exceed the threshold for firing a spike and the classification accuracy will increase consequently.

Compared with $\Delta \omega_{i}$ in (4), Tempotron-VR multiplies an additional factor which is correlated with the maximal membrane voltage $V_{mn}^{max}$ of the *n*th neuron in the group *m*. To obtain the factor, we need to know the ratio of $V_{tn}^{max}$ (*t* denotes the target group) to the sum of all groups $\sum_{m}V_{mn}^{max}$. To ensure the ratio positive, we transform $V_{mn}^{max}$ by:

(5) $$f\left(V_{mn}^{max}\right)=ln\left(e^{V_{mn}^{max}}+1\right).$$

We use $F(V_{mn}^{max})$ to represent the ratio of $f(V_{tn}^{max})$ to the sum of all groups $\sum_{m}f(V_{mn}^{max})$:

(6) $$F\left(V_{mn}^{max}\right)=ln\left(\frac{f(V_{tn}^{max})}{\sum_{m}f(V_{mn}^{max})}\right).$$

The derivative of $F(V_{mn}^{max})$ for neurons in the target group *t* and other groups can be computed by:

(7) $$F^{\prime }\left(V_{mn}^{max}\right)=f^{\prime }\left(V_{mn}^{max}\right)\cdot \left(\frac{1}{f(V_{mn}^{max})}-\frac{1}{\sum_{m}f(V_{mn}^{max})}\right),m=t,$$

(8) $$F^{\prime }\left(V_{mn}^{max}\right)=f^{\prime }\left(V_{mn}^{max}\right)\cdot \left(-\frac{1}{\sum_{m}f(V_{mn}^{max})}\right),m\neq t.$$

Finally we multiply $\Delta \omega_{i}$ by $F^{\prime }\left(V_{mn}^{max}\right)$ to obtain $\Delta \omega_{i}^{\prime }$:

(9) $$\Delta \omega_{i}^{\prime }=\lambda \sum_{t_{i}<t_{max}}K\left(t_{max}-t_{i}\right)\cdot F^{\prime }\left(V_{mn}^{max}\right)$$

While $\omega_{ij}$ is updated by $\Delta \omega_{i}^{\prime }$ at each iteration, $V_{mn}^{max}$ of neurons in the target group becomes larger (due to $F^{\prime }\left(V_{mn}^{max}\right)$ greater than 0) and $V_{mn}^{max}$ of others becomes smaller (due to $F^{\prime }\left(V_{mn}^{max}\right)$ smaller than 0) during training process, which leads to the neurons in the target group firing spikes more easily and others firing no spike. This strategy helps to prevent over fitting and improves recognition performance.

During the process of training, for neurons in group *m* which represents the *m*th odor type, we use training samples with class label *m* as positive samples and others as negative samples. During the process of testing, we use the majority voting method to predict the most likely odor type. By counting the firing neurons (neuron that fires a spike) in each group, the class represented by the group which has the maximal number of firing neurons is the predicted odor type.

## 3. Experimental Results

In this section, comprehensive experiments are conducted to demonstrate the effectiveness of our approach in odor recognition tasks. Firstly, we present the odor recognition performance of SNN-VR in comparison with other approaches. Secondly, we compare the SNN-VR approach with typical bin-based methods to show the effectiveness of adopting precise timing information. Thirdly, we compare SNN-VR approach with classical SNN model to demonstrate the strengths of the voltage-based regulation strategy in neural signals. After that, we investigate the recognition performance under different time periods to show the ability of SNN-VR for quick recognition. Finally, we briefly discuss the pros and cons of SNN-VR.

### 3.1. Odor Recognition Performance

In this experiment, we present the odor recognition performance of Tempotron-VR in comparison with Tempotron and bin-based approaches. Considering the odor stimulation time is 4–5 s, we choose the 5-s-long spike trains to fully use the information of neural signals in olfactory bulb during odor stimulation. For the model parameters, all the parameters in the method are selected with cross validation. In each experimental trial, 20% of the training set is used for validation. For KNN, we select the number of nearest neighbors. For SVM, we select both parameters of C and gamma. For SNN, we choose the τ and $\tau_{s}$ in Tempotron and Tempotron-VR respectively. For the bin-based classifiers, the bin size is set to 0.5 s. The results are shown in Table 3.

As shown in Table 3, we present the classification accuracy for two-class, three-class and four-class classification tasks respectively. Overall, the proposed Tempotron-VR method achieves the highest performance for odor recognition.

Compared with bin-based approaches, the SNN models obtain higher recognition accuracy. In bin-based classifiers, MLE achieves the highest recognition accuracy, while Tempotron-VR shows similar (two-class 82%∖83%) and better (three-class 71%∖69% & four-class 63%∖57%) performance than MLE. Since the SNN models do not rely on bin-based inputs, they better exploit the temporal information in spike trains, which leads to higher decoding performance.

Compared with Tempotron, the Tempotron-VR approach obtains better recognition performance. As shown in Table 3, the recognition accuracy of Tempotron-VR is significantly higher than that of Tempotron. More specifically, the average recognition accuracy of two-class, three-class and four-class of Tempotron-VR is 8%, 14% and 15% higher than that of Tempotron respectively. Since the training dataset for odor recognition is small, the voltage-based regulation strategy can learn more effectively and improves odor recognition performance.

### 3.2. Comparison with Bin-Based Methods

**Figure 3:**
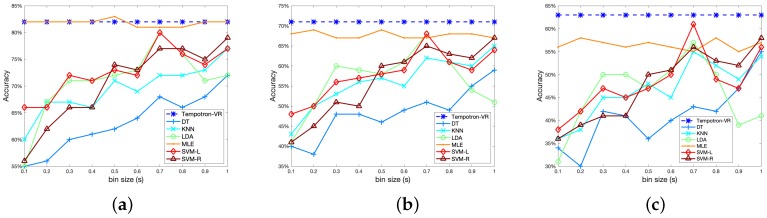
Odor recognition accuracy using different bin sizes: (**a**) two-class case; (**b**) three-class case; (**c**) four-class case. The horizontal axis represents the bin size.

In this experiment, we compare our method with bin-based approaches using different bin sizes. For bin-based classifiers, bin size is a crucial parameter that influences the odor recognition performance. Therefore, we evaluate the bin-based classifiers with different settings of bin sizes, and the results are shown in Figure 3. Figure 3a–c illustrate the results for two-class, three-class and four-class tasks, respectively.

As shown in Figure 3, the optimal choice of bin size varies among different classifiers. For classifiers such as DT, KNN, and SVM-R, the odor recognition performance increases as we use larger bin sizes. For LDA and SVM-L, the odor recognition accuracies firstly improve with the increase of bin size, and then fall after a peak at a bin size of 0.7. For the MLE classifier, the influence of bin size is small, and slightly better performance can be obtained using small bin sizes below 0.5. For most of these classifiers, the accuracies are highly sensitive to the choice of bin size. However, using small training data, finding an optimal bin size is usually difficult. In addition, the optimal bin size can be different for different tasks.

Compared with bin-based classifiers, one advantage of SNN is that it can directly input the time points of spike trains, which avoids the bin size selection process. As shown in Figure 3, the proposed Tempotron-VR method achieves the highest performance compared with bin-based classifiers using different bin sizes. The Tempotron-VR method is more feasible and effective in neural signal-based odor recognition tasks.

### 3.3. Effectiveness of Voltage-Based Regulation Strategy

In this experiment, we evaluate the odor recognition performance of Tempotron-VR with different training set sizes. Since data acquisition of neural signals is expensive, the training dataset is usually small, which can cause an overfitting problem in Tempotron-based model learning. In Tempotron-VR, the voltage-based regulation strategy strengthens synaptic weights to enhance the maximal membrane voltage of neurons in the target group at each iteration and improves recognition performance.

**Figure 4:**
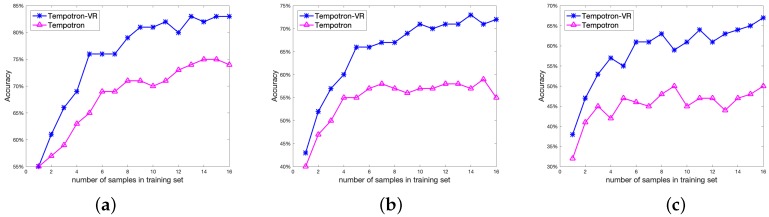
Odor recognition accuracy using different training sets: (**a**) two-class case; (**b**) three-class case; (**c**) four-class case. The blue line and red line represent Tempotron-VR and Tempotron algorithms, respectively. The horizontal axis represents the number of sample groups in training sets (each sample group contains a sample from each class).

To demonstrate the strengths of the proposed voltage-based regulation strategy, we compare Tempotron-VR with Tempotron using different training data sizes. We tune the number of training sample groups from 1 to 16, where each sample group contains one sample from each class. The sample groups are randomly shuffled. The rest of the samples are used for testing. The results are shown in Figure 4a–c, for two-class, three-class, and four-class, respectively.

As shown in Figure 4, the odor recognition accuracies of Tempotron-VR are higher than Tempotron over all the settings. For the two-class task, the classification accuracies of Tempotron-VR are 76%, 81%, and 82% using 6, 10, and 14 training sample groups, which are 7%, 11%, and 7% higher than the Tempotron approach. For the three-class task, Tempotron-VR’s accuracies with 6, 10, and 14 training sample groups are 66%, 71%, and 73%, which outperforms Tempotron by 9%, 14%, and 16%, respectively. For the four-class task, the improvement is more significant. With 6,10, and 14 training sample groups, the Tempotron-VR obtains accuracies of 61%, 61%, and 64%, which improved by 15%, 16%, and 17%, compared with Tempotron. It is also notable that, with only 10 training sample groups, the average accuracy of Tempotron-VR can reach 82%, 70% and 64% for two-class, three-class and four-class odor recognition, respectively, which demonstrates high learning performance with small training data.

### 3.4. Performance of Quick Recognition

**Figure 5:**
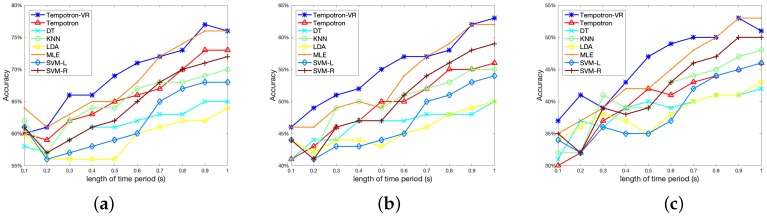
Odor recognition accuracy within short time periods: (**a**) two-class case; (**b**) three-class case; (**c**) four-class case. The horizontal axis represents the length of time periods.

In BMI-based bioelectronic noses, the response time is an important criterion. In this experiment, we test the recognition performance of our method under different time periods (from 0.1 s to 1 s), to evaluate the performance for quick recognition. The results are shown in Figure 5.

As shown in Figure 5, the odor recognition performance improves as we tune the time period from 0.1 s to 1 s. With the time period of 1 s, the Tempotron-VR achieves accuracies of 77%, 63%, and 53% in two-class, three-class and four-class odor recognition tasks, respectively. With a response time of 0.5 s, the accuracies of Tempotron-VR are 69%, 55%, and 47%. The proposed Tempotron-VR method demonstrates good odor recognition ability within a short time period and is promising for quick detection in bioelectronic noses.

### 3.5. Discussion

Experimental results show that the proposed Tempotron-VR approach achieves the state-of-the-art performance compared with bin-based classifiers and Tempotron. The results also demonstrate the advantages of Tempotron-VR. Compared with bin-based classifiers, our method avoids the selection of bin size by directly using spike timings as input, which make it more feasible for practical applications. In addition, the timing information of spikes can be better exploited to achieve higher odor recognition performance. Compared with Tempotron, the proposed Tempotron-VR strategy can learn more effectively from small training data, which is crucial in neural signal-based odor decoding where signal acquisition can be extremely expensive.

One drawback of SNN-based odor recognition is that the computational cost of SNNs is relatively higher. We evaluate the time cost of each experimental trial in the training stage and the run time of each sample in test stages with SNN and SNN-VR. The experiments are carried out on a desktop computer with an Intel(R) Core(TM) i5-4590 CPU, 8 GB RAM memory and 64 bit operating system. In an experimental trial, for each odor type, 16 samples are randomly selected for training, and the four remaining samples are used for testing. It is repeatedly executed ten times for measuring the average results and the time is measured in seconds. As shown in Table 4, SNN costs 10.77 s, 19.37 s and 27.63 s for two-class, three-class and four-class tasks respectively. For SNN-VR, the average training time of each trial is slightly higher, which is 13.94 s, 21.04 s and 28.57 s respectively. To predict a test sample, SNN needs 0.011 s, 0.012 s and 0.011 s on average for two-class, three-class and four-class tasks, respectively, while, in SNN-VR, the average testing time of each sample is 0.019 s, 0.020 s and 0.021 s, respectively. The results show that, although the computational cost of the SNN-VR method is relatively higher, the online test time for each sample is only around 0.02 s, which is acceptable in applications. In addition, this problem is expected to be solved by using specialized hardware for SNN computing, i.e., the neuromorphic chips [34]. The specialized chips contain millions of neurons, which make it possible to compute SNN models in real time [34]. These chips are computationally efficient and energy-saving [35], which is promising for applications in bioelectronic noses.

## 4. Conclusions

In this study, we propose a novel spiking neural network (SNN)-based neural signal decoder to recognize odors with spike train signals recorded from the main olfactory bulb in rats. The proposed SNN-based approach exploits rich timing information well in precise time points of spikes. To deal with small training data, we especially propose a new SNN learning method with a voltage-based regulation strategy (Tempotron-VR), in order to alleviate the overfitting problem. Results show that, compared with bin-based odor decoders, the SNN approaches achieve better performance in two-class, three-class, and four-class odor recognition tasks. With the proposed voltage-based regulation strategy, the SNN-VR approach obtains about 15% improvement compared with classical SNN models, while SNN-VR demonstrates better generalization ability especially with small training sets.

## Figures and Tables

**Table 1:** Summary of bin-based approaches.

Study	Method	Task	Bin Size	Neuron	Feature
[17]	Maximum likelihood estimation	odor recognition	0.1 s	128	bin-based vector
[12]	Population vector similarity	low concentration odor detection	0.1–1.0 s	10	
[18]	Kalman filter	lower limb muscle activity prediction	0.03 s	16	

**Table 2:** Description of neural signals.

Content	Value
Rat	1
Odor types	4
Recorded neurons	11
Samples per odor	20
Recording time length	40 s
Stimulation time length	4–5 s

**Table 3:** Odor recognition accuracy with SNNs and bin-based classifiers.

Odor Combination	DT	KNN	LDA	MLE	SVM-L	SVM-R	Tempotron	Tempotron-VR
1 2	65%	74%	90%	97%	92%	89%	86%	95%
1 3	62%	78%	63%	93%	63%	79%	68%	81%
1 4	65%	71%	71%	92%	72%	76%	68%	79%
2 3	64%	71%	75%	79%	77%	71%	77%	92%
2 4	71%	82%	91%	92%	90%	76%	90%	97%
3 4	43%	47%	41%	47%	44%	51%	57%	50%
Avg	62%	71%	72%	83%	73%	74%	74%	82%
1 2 3	52%	64%	66%	80%	63%	68%	63%	82%
1 2 4	50%	65%	72%	83%	73%	72%	60%	84%
1 3 4	40%	50%	44%	57%	44%	51%	45%	55%
2 3 4	41%	50%	51%	54%	52%	49%	59%	64%
Avg	46%	57%	58%	69%	58%	60%	57%	71%
1 2 3 4	36%	48%	47%	57%	47%	50%	48%	63%

**Table 4:** Training and test time of SNN and SNN-VR.

Number of Odors	Training		Test	
	SNN	SNN-VR	SNN	SNN-VR
2	10.77	13.94	0.011	0.019
3	19.37	21.04	0.012	0.020
4	27.63	28.57	0.011	0.021

## Abbreviations

The following abbreviations are used in this manuscript:

BMI	Brain–Machine Interface
SNN	Spiking Neural Network
LIF	Leaky Integrate and Fire
DT	Decision Tree
KNN	k-Nearest Neighbors
LDA	Linear Discriminant Analysis
MLE	Maximum Likelihood Estimation
SVM-L	Support Vector Machine (Linear kernel)
SVM-R	Support Vector Machine (RBF kernel)
PCA	Principal Component Analysis
SNN-VR	SNN with Tempotron-VR
Tempotron-VR	Tempotron with Voltage-Based Regulation
